# HAMLET, a human milk protein-lipid complex, modulates amoxicillin induced changes in an *ex vivo* biofilm model of the oral microbiome

**DOI:** 10.3389/fmicb.2024.1406190

**Published:** 2024-07-04

**Authors:** Navdeep Kaur Brar, Achal Dhariwal, Sudhanshu Shekhar, Roger Junges, Anders P. Hakansson, Fernanda Cristina Petersen

**Affiliations:** ^1^Institute of Oral Biology, Faculty of Dentistry, University of Oslo, Oslo, Norway; ^2^Institute of Experimental Infection Medicine, Faculty of Medicine, Lund University, Malmö, Sweden

**Keywords:** antibiotic resistance, HAMLET, oral microbiome, oral resistome, amoxicillin, probiotics

## Abstract

Challenges from infections caused by biofilms and antimicrobial resistance highlight the need for novel antimicrobials that work in conjunction with antibiotics and minimize resistance risk. In this study we investigated the composite effect of HAMLET (human alpha-lactalbumin made lethal to tumor cells), a human milk protein-lipid complex and amoxicillin on microbial ecology using an *ex vivo* oral biofilm model with pooled saliva samples. HAMLET was chosen due to its multi-targeted antimicrobial mechanism, together with its synergistic effect with antibiotics on single species pathogens, and low risk of resistance development. The combination of HAMLET and low concentrations of amoxicillin significantly reduced biofilm viability, while each of them alone had little or no impact. Using a whole metagenomics approach, we found that the combination promoted a remarkable shift in overall microbial composition compared to the untreated samples. A large proportion of the bacterial species in the combined treatment were *Lactobacillus crispatus*, a species with probiotic effects, whereas it was only detected in a minor fraction in untreated samples. Although resistome analysis indicated no major shifts in alpha-diversity, the results showed the presence of *TEM* beta-lactamase genes in low proportions in all treated samples but absence in untreated samples. Our study illustrates HAMLET’s capability to alter the effects of amoxicillin on the oral microbiome and potentially favor the growth of selected probiotic bacteria when in combination. The findings extend previous knowledge on the combined effects of HAMLET and antibiotics against target pathogens to include potential modulatory effects on polymicrobial biofilms of human origin.

## Introduction

The human oral microbiome is considered the second-largest microbial community, following the gut microbiota, in terms of both diversity and complexity (Wade, 2013; Caselli et al., 2020). A broad range of odontogenic inflammatory infections, from periodontitis and peri-implantitis to post-traumatic osteomyelitis and facial cellulitis, have been associated with oral biofilms featuring polymicrobial communities (Hajishengallis and Lamont, 2000; Gaetti-Jardim et al., 2012; Dashper et al., 2019; Anju et al., 2022; Masters et al., 2022; Costa et al., 2023). These biofilms are difficult to treat because of their intrinsic antibiotic tolerance and resistance to the host’s immune system. Additionally, the oral cavity harbors one of the highest abundance of antibiotic resistance genes (ARGs) in the entire human body, surpassing the abundance of ARGs in the gut (Carr et al., 2020; Maestre-Carballa L et al., 2022). As such, the oral cavity serves as a significant potential source for the dissemination of antibiotic resistance (Roberts and Mullany, 2010; Anderson et al., 2023). Combining different therapies with potential antimicrobial activities has emerged as a novel strategy to overcome the challenges posed by polymicrobial infections (Shrestha et al., 2022). One particular combination that has demonstrated promising results is the utilization of HAMLET (Human Alpha-lactalbumin Made Lethal to Tumor Cells), a protein-lipid complex, in conjunction with antibiotics.

HAMLET is a complex comprised of alpha-lactalbumin and oleic acid that has demonstrated potent cancer cell-killing capabilities, while sparing healthy, differentiated cells, rendering it a promising potential therapeutic (Håkansson et al., 1995; Svensson et al., 2000, 2002). Additionally, HAMLET exhibits antimicrobial properties against key human pathogens. Although mostly active against Gram-positive bacteria, such as *Streptococcus pneumoniae* and *Streptococcus pyogenes*, HAMLET has also shown bactericidal effects on selected Gram-negative species, such as *Haemophilus influenzae* and *Moraxella catarrhalis* (Marks et al., 2012). Notably, HAMLET’s bactericidal activity has not been detected in other Gram-negative pathogens, including *Escherichia coli*, *Klebsiella pneumoniae*, *Pseudomonas aeruginosa*, *Haemophilus parainfluenzae*, and *Enterobacter cloacae* (Håkansson et al., 2000; Hakansson et al., 2011; Marks et al., 2012, 2013; Alamiri et al., 2019; Meikle et al., 2019; Roche-Hakansson et al., 2019). When used in combination with antibiotics, HAMLET has demonstrated the ability to lower the minimum inhibitory concentration (MIC) of methicillin for methicillin-resistant *Staphylococcus aureus* (MRSA) strains, bringing them within the sensitive range. HAMLET augment also the efficacy of selected antibiotics against antibiotic-resistant bacterial strains such as *S. pneumoniae* and *Mycobacterium tuberculosis* (Marks et al., 2012, 2013; Meikle et al., 2019).

Among the most commonly prescribed antibiotics in primary healthcare settings and for odontogenic infections is amoxicillin (Akhavan and Vijhani, 2023). This broad-spectrum *β*-lactam antibiotic is a modified form of penicillin with an extra amino group. Its mechanism of action involves disrupting peptidoglycan cross-linking in the bacterial cell-wall. Amoxicillin inactivates and kills pathogens by binding to penicillin-binding-proteins (PBPs) located on the bacterial membrane (Akhavan and Vijhani, 2023; National Center for Biotechnology Information, 2023; Akhavan et al., 2024). However, its efficacy against polymicrobial biofilms such as in the oral cavity can be limited due to a number of factors, including the formation of a protective barrier that can prevent antibiotics from effectively reaching and killing the bacteria within the biofilms. Further, the production of beta-lactamases by members of microbial communities can reduce the concentration of active amoxicillin available. In combination with HAMLET, other beta-lactam antibiotics have shown synergistic effects against both *S. pneumoniae* and *S. aureus MRSA* biofilms (Marks et al., 2012, 2013). This suggests that the inclusion of HAMLET in combination with amoxicillin may have potential as an effective strategy for treating polymicrobial biofilms.

In this study, we used an *ex vivo* oral microbiome model to provide a relevant testbed for investigating the effects of HAMLET and amoxicillin on microbial ecology. Our findings indicate that the combination of amoxicillin and HAMLET act together to inhibit bacterial viability in polymicrobial biofilms. Furthermore, the combination at low concentrations influenced the microbial ecology of the oral microbiome, leading to a proportional increase in bacterial species exhibiting probiotic characteristics.

## Methods

### Sample collection

The research followed the ethical principles directed in the Declaration of Helsinki and received approval from the National Regional Ethics Committee (REK20152491) for studies involving human samples. Eight participants, age group (25–35 years) were instructed to brush their teeth following breakfast and to abstain from food or drink for a minimum of 2 h before providing saliva samples. Additionally, they rinsed their mouths three times with water, 10 min before saliva collection. The participants were healthy and none of them were using any medicines or had used antibiotics in the last 6 months. Non-stimulated saliva was collected, and these samples were centrifuged at 6,000 × *g* for 5 min at 4°C. This centrifugation step effectively precipitated larger debris and eukaryotic cells. The resulting supernatant was pooled and utilized as the inoculum in the human oral microbiome biofilm model, as described below.

A second centrifugation was conducted to obtain cell-free saliva by spinning down the samples at 10,000 × *g* for 7 min at 4°C. The upper fraction was used to coat the bottom of the wells prior to biofilm growth in a process termed as ‘pellicle formation’ to mimic the establishment of an oral biofilm (Edlund et al., 2013).

### HAMLET production

HAMLET was produced in three steps: (1) purification of alpha-lactalbumin from human milk, (2) converting native alpha-lactalbumin to partially unfolded protein in the presence of oleic acid (C18:1) and (3) dialysis and lyophilization as previously described (Håkansson et al., 2000; Svensson et al., 2000).

### The human oral microbiome biofilm model

We utilized a previously established *ex vivo* biofilm model designed to preserve a highly reproducible diversity of species and metabolic activity within the human oral microbiome (Edlund et al., 2013, 2018). In summary, SHI media was pre-reduced for 4 h under anaerobic conditions, characterized by a carbon dioxide level of 5%, balanced with nitrogen. SHI media was prepared as previously described (Tian et al., 2010). The pooled saliva samples were added at a ratio of 2 μL of saliva per mL SHI medium. These were allotted into the wells of a 24-well plate, with each well containing 1 mL of the mixture. The plate was then incubated within an anaerobic chamber at 37°C for 24 h.

After this incubation period, the supernatant was removed and replaced with fresh SHI medium to support the pre-formed oral biofilms. In the first set of experiments, the samples were either left untreated (control), or treated with amoxicillin ranging from 0 to 200 μg mL^−1^ (Sigma-Aldrich). In the second set of experiments, the preformed biofilms were not treated (control), treated with amoxicillin 0.1 μg mL^−1^, HAMLET ranging between 125 and 250 μg mL^−1^, or with a combination treatment composed of HAMLET ranging between 125 and 250 μg mL^−1^ in conjunction with amoxicillin at 0.1 μg mL^−1^. The stock solution of amoxicillin (2 mg/mL in distilled water) and HAMLET [5 mg/mL in phosphate-buffered saline (PBS)] were appropriately diluted in SHI medium before adding to the biofilms.

Following an incubation period of another 24 h, the oral biofilms were washed with PBS, followed by suspension in 1 mL of PBS. Glycerol (20%) was added to the samples before they were archived and stored at −80°C.

### Oral biofilm viability assay

To evaluate the viability of the biofilms, samples obtained from both the control and the treatment groups, were subjected to a 10-fold dilution series. Subsequently, 20 μL of each dilution was plated onto SHI agar plates. These plates were then incubated for 48 h at 37°C within an anaerobic chamber. Then the number of colony forming units per milliliter (CFUs mL^−1^) was calculated, and represented as log 10-transformed values.

### Biofilm biomass

To evaluate HAMLET alone, amoxicillin alone or HAMLET-amoxicillin combination impact on oral microbiome biomass, we utilized a dry weight measurement procedure. A portion of the biofilm suspension was allocated into pre-weighed tubes and mixed with thrice the volume of absolute ethanol, followed by chilling at −20°C for 20 min. Centrifugation at 10,000 × *g* for 5 min at 4°C was then performed, after which the supernatant was discarded. The biofilms were dried with heat and vacuum, and dry weight was determined by weighing the tubes before and after the process.

### Real-time PCR

To quantify bacterial DNA, universal 16S rRNA primers FP1067 (5′ CCATGAAGTCGGAATCGCTAG) and FP1068 (5′ GCTTGACGGGCGGTGT) were employed as detailed by Yigit et al. (2016). Duplication was ensured for all the reactions. A 25 μL PCR reaction was set up, including 12.5 μL of Maxima SYBR Green/ROX qPCR Master Mix (2×), encompassing Maxima Hot Start Taq DNA Polymerase, deoxynucleotide triphosphates (dNTPs), and SYBR Green I dye within a ROX-supplemented PCR buffer, 0.4 μM of each primer, and 1 μL of the DNA template, not exceeding 70 ng. The mix was brought to volume with nuclease-free water. The PCR protocol involved an initial denaturation for 10 min at 95°C, followed by a 40-cycle amplification process—denaturation at 98°C for 30 s, annealing at 55°C for 60 s, and elongation at 72°C for 60 s.

### DNA extraction

Bacterial DNA was extracted using the MasterPure™ Gram Positive DNA Purification Kit (Epicentre, Madison, WI, United States), following the manufacturer’s established protocol. Subsequently, the precipitated DNA was resuspended in 35 μL milliQ water. To assess the quality and quantity of the extracted DNA, NanoDrop^TM^ 2000c spectrophotometer (Thermo Fisher Scientific, Waltham, MA, United States) was used for initial evaluation. This was followed by quantification using Qubit TM 4 Fluorometer (Thermo Fisher Scientific, Waltham, MA, United States) to yield precise measurements of the DNA’s concentrations.

### DNA library preparation and sequencing

The preparation of the DNA libraries was executed with the Illumina DNA Prep (M) kit, (Illumina, Inc., San Diego, CA, United States), in strict adherence to the manufacturer’s protocol. To assess the quality and concentration of the DNA library, initial measurements were conducted using the NanoDrop™ 2000c spectrophotometer and Qubit™ 4 Fluorometer. Finally, analysis involved the utilization of a Bioanalyzer (Agilent Technologies, Santa Clara, CA, United States) using a High Sensitivity DNA kit (Agilent Technologies, Santa Clara, CA, United States).

The DNA library was obtained by resuspending it in the provided buffer. Each sample was adjusted to 500 ng DNA in a 30 μL volume using nuclease-free water.

For the metagenomic shotgun sequencing approach, services at the Norwegian Sequencing Centre (Oslo, Norway) were utilized, using the Illumina NovaSeq 6,000 SP platform (Illumina, Inc., San Diego, CA, United States). The paired-end sequencing reads were generated with a corresponding read length of 150 base pairs.

### Assessment of sequencing read quality

The evaluation of sequencing read quality, both in raw and preprocessing state, was conducted utilizing FastQC tool (v.0.11.9) (Andrews, 2010). The identification and removal of low-quality reads, as well as the elimination of adapter sequences, was achieved using Trimmomatic (v.0.39). The following parameters were used during this process: ILLUMINACLIP: Nextera PE:2:30:10 LEADING:3 TRAILING:3 SLIDING WINDOW:4:15 MINLEN:36. The remaining high-quality reads were subjected to microbiome and resistome profiling.

### Taxonomic and resistome profiling

MetaPhlAn3 software (v.3.7.0) (Truong et al., 2015) was used to profile the bacterial composition in the oral biofilm samples and to determine their abundance at species-level using default settings. The ‘*merge metaphlan tables.py*’ script was used to merge the profiled metagenomes into an abundance table. To detect the hits to known Antibiotic Resistance Genes (ARGs), “high quality” paired-end reads were mapped against the Comprehensive Antibiotic Resistance Database (CARD) (v.3.2.2) (Alcock et al., 2020; Database TCAR, 2020) by using the KMA alignment tool (1.4.12) (Clausen et al., 2018) with parameters: *-ipe*, *-tmp*, -*1t1*, -*and*, -*apm f*, -*ef*. The list of detected ARGs was filtered to include only those with a minimum threshold of 80% identity between the query and reference gene over at least 80% of the reference gene length.

### Downstream analysis

Two key software tools were used to conduct comprehensive exploration, analysis, and visualization of the microbiome and resistome count data: MicrobiomeAnalyst (Dhariwal et al., 2017; Chong et al., 2020) and ResistoXplorer (Dhariwal et al., 2021).

For graphical representation and statistical analysis, GraphPad Prism (Prism 9 and 10 software) as well as the R programming (version 4.2.1) were utilized. Alpha-diversity was calculated using the Shannon and Chao1 diversity indexes at species level, as well as ARG level. For Beta-diversity, Aitchison distance metric on centered log-ratio (CLR) transformed counts were executed by phyloseq R package. The resulting data was visualized in compositional principal component analysis (PCA) ordination plot.

The top 10 most abundant features of the microbiome (species) and resistome (ARGs) data were plotted using *aggregate top taxa* and plotting functions of the microbiome R package.

Pairwise comparisons of log-fold changes in the abundance of microbial species and ARGs between different groups were performed using DESeq2 (Love et al., 2014). In order to account for multiple testing, Benjamini–Hochberg (BH) procedure was employed to adjust the results (adjusted *p*-values).

In case where “one-way analysis of variance” (ANOVA) was conducted, the results were adjusted for multiple comparisons using the Dunnett’s multiple comparison test. Adjusted *p*-values lower than 0.05 were considered statistically significant.

## Results

### Dose-dependent effects of amoxicillin on oral biofilms

**Figure 1:**
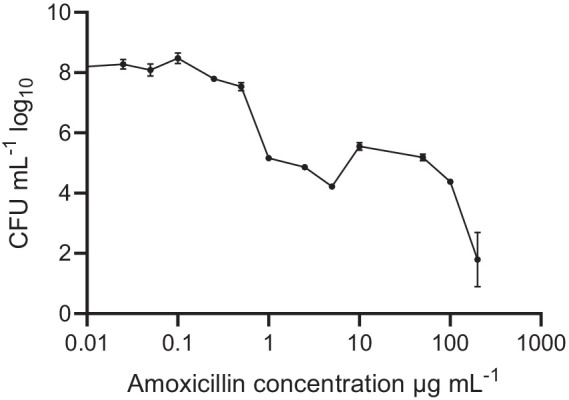
Oral biofilm treated with different amoxicillin concentrations. Number of viable cells in the polymicrobial biofilm community, as determined by colony-forming units, counted on SHI agar plates. The data are shown for triplicate experiments as mean ± SE. For visualization purposes, the untreated samples were given a value of 0.01 to adjust it to the log-scale. All concentrations > or = 1 μg mL^−1^ were significantly different from the untreated control, using One-way ANOVA followed by Dunnett’s multiple comparison test.

Pre-formed oral biofilms in new fresh SHI media were initially subjected to varying concentrations of amoxicillin, ranging from 0 to 200 μg/mL (Figure 1). The inoculum was prepared using pooled saliva obtained from eight donors. Notably, when exposed to low amoxicillin concentration within the range of 0.025–0.1 μg/mL, no significant effects were observed. However, as the amoxicillin concentration exceeded 0.5 μg/mL, a contrasting effect was observed (*p* < 0.001) where biofilm viability was gradually inhibited. The reduction in viability continued until the highest amoxicillin concentration was reached, at which point viable cells were almost undetectable.

### The impact of HAMLET and low concentrations of amoxicillin on oral biofilms

**Figure 2:**
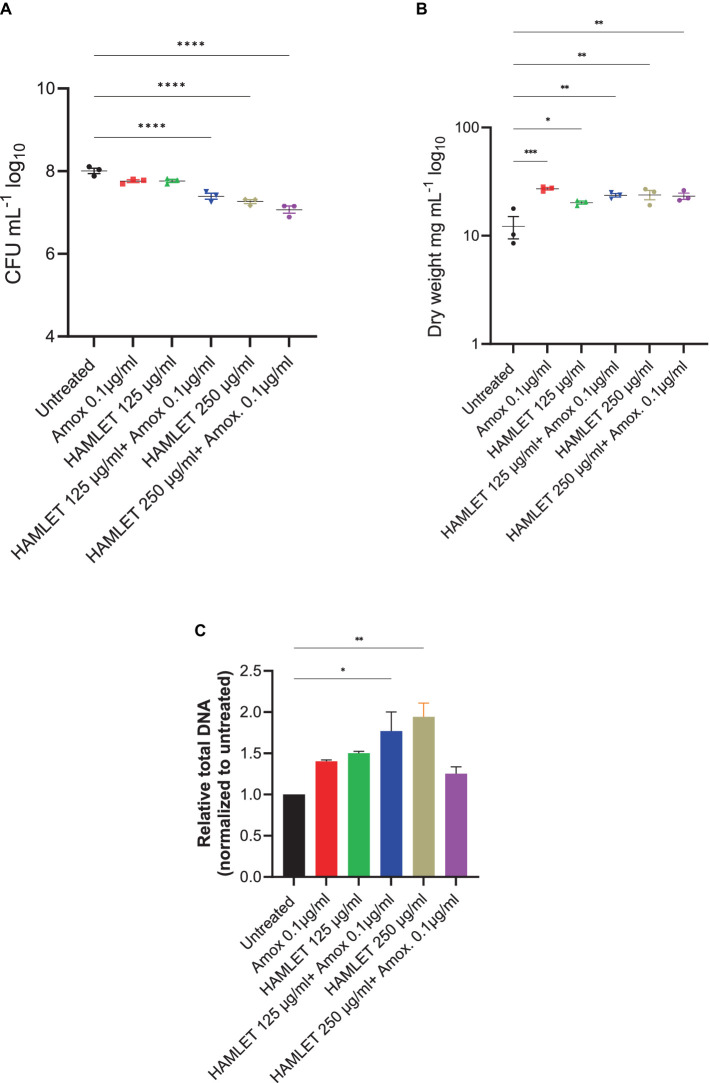
The effect of HAMLET alone or in combination with low amoxicillin concentration on oral biofilm community. **(A)** Numbers of viable cells in the community are determined by colony-forming units. **(B)** Biomass determined by dry weight. **(C)** Total DNA determined by RT-PCR using universal primers for the 16S rRNA gene, normalized to untreated. **(A,B)** The results are based on three independent experiments with triplicate samples. **(C)** The results are based on two independent experiments with triplicate samples. Data are shown as mean ± SE. *p < 0.05, **p < 0.01, ***p < 0.001, ****p < 0.0001. One-way ANOVA followed by Dunnett’s multiple comparison test.

To evaluate the impact of HAMLET, both as a standalone treatment and in combination with amoxicillin at low concentrations, pre-formed oral biofilms were subjected to two different HAMLET concentrations, alone or in combination with 0.1 μg/mL of amoxicillin for 24 h. We started with a concentration of 250 μg/mL, as minimal bactericidal effects of HAMLET at this level against specific bacterial species have been previously reported (Marks et al., 2012). A reduction in CFU was observed when HAMLET was used alone and in combination with amoxicillin at this concentration, exhibiting an inhibition by 0.75 and 0.93 log units, respectively (Figure 2A). We then tested HAMLET at 125 μg/mL. At this concentration neither HAMLET alone nor amoxicillin alone, when assessed in comparison to the negative control, displayed any significant reduction in bacterial cell viability. However, oral biofilm viability showed to be significantly affected by the combination treatment of HAMLET at 125 µg/mL and 0.1 μg/mL amoxicillin, leading to significant decrease in bacterial viability compared to untreated samples by 0.61 log (Figure 2A). We chose this HAMLET concentration for further metagenomic analysis to highlight the potential synergistic effects of the combination with amoxicillin. Compared to the untreated control, there was a significant increase in dry biomass for all the treatment groups (Figure 2B). However, The total DNA load was only significantly increased in biofilm treated with 250 μg/mL HAMLET alone and in combination treatment 125 μg/mL HAMLET + amoxicillin 0.1 μg/mL (Figure 2C).

### Impact of HAMLET alone or in combination with amoxicillin oral microbiome ecology

A total of eight samples, representing two samples from each treatment group, underwent shotgun metagenomic sequencing. This analysis resulted in the generation of approximately 90.2 million paired reads after quality filtering, yielding an average of 11.3 million reads (with a minimum of 6.8 million and a maximum of 18.5 million reads per sample).

**Figure 3:**
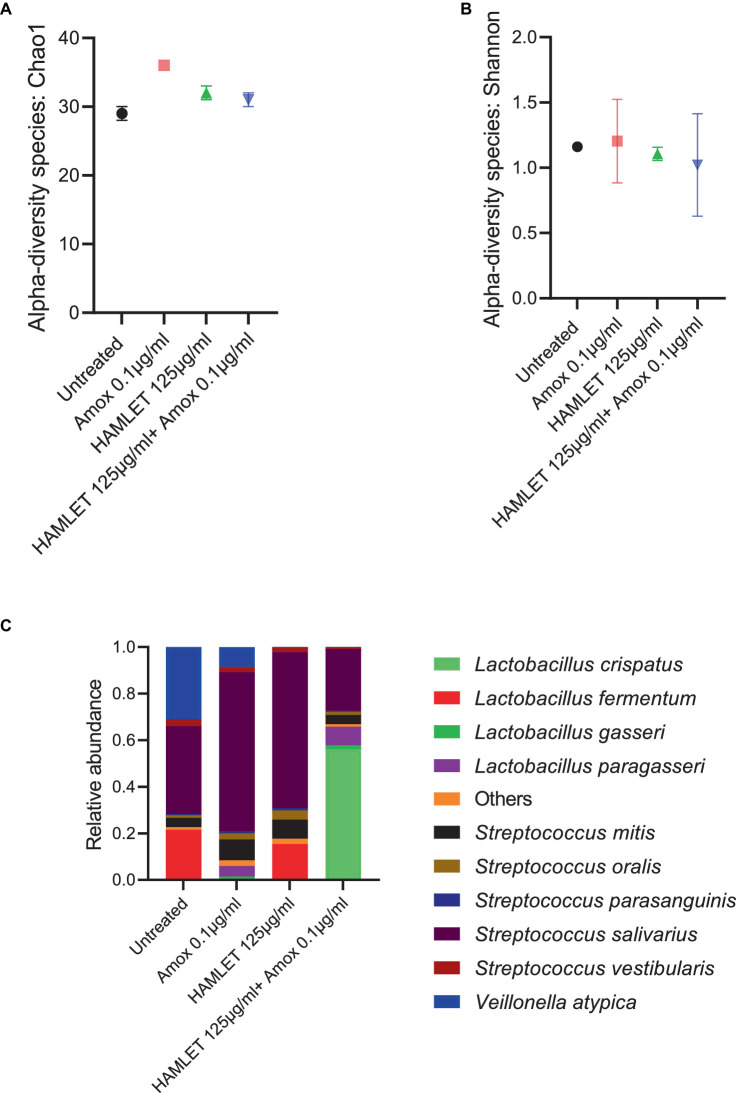
Ecological impact of HAMLET alone or in combination with amoxicillin on oral biofilm community. **(A,B)** Alpha-diversity on species level measured by **(A)** Chao1 index indicate the total richness and **(B)** Shannon index indicate richness and evenness. **(C)** Stacked bar plots illustrate the relative abundance of all replicates for the 10 most abundant species. All results are based on two biological replicates from the same day. All results are based on two biological replicates from the same day.

The metagenomic analysis provided insights into the effect of HAMLET, used either as a standalone treatment or in combination with amoxicillin, on the ecology of the oral microbiome. To assess changes in alpha diversity, metrics such as the Chao1 index, which quantifies only microbial richness, and the Shannon index, which accounts for both richness and evenness (abundance), were employed. At species level, no significant changes in either alpha diversity index were observed in treatment samples when compared to the untreated control (Figures 3A,B).

In total, 44 bacterial species spanning eight bacterial genera across all the samples were identified (Supplementary Table S1). Despite biological sample variation, alterations in the relative abundance of taxonomic composition were evident in the treatment groups compared to the negative control (Supplementary Table S2). Beta diversity analysis revealed increased variability in the biological replicates subjected to antibiotic treatment, contrasting with the biological replicates from untreated or HAMLET solely treated samples (Supplementary Figure S1).

Analyzing the taxonomic composition at the species level revealed the emergence of new species in the amoxicillin, HAMLET and HAMLET combined with amoxicillin treated biofilm groups (Figure 3C). In comparison to the untreated samples, *Streptococcus salivarius* emerged as the dominant species, while *Lactobacillus fermentum* decreased significantly in the amoxicillin treated group (Supplementary Figure S1). Both *L. fermentum* and *S. salivarius* reduced in proportion when subjected to the combination of HAMLET and amoxicillin. In contrast, *Lactobacillus crispatus* increased significantly in proportion and dominated in the combination treatment group (Supplementary Figure S1).

Furthermore, the presence of the pathogen *Streptococcus pneumoniae* was noted in all samples, with an increase in amoxicillin-treated samples compared to the untreated control (Supplementary Table S1).

### Impact of HAMLET alone or in combination with amoxicillin on the resistome

Across all samples, a total of 123,350 paired reads were annotated as ARGs. On average, there were 15,418 reads per sample, with a minimum count of 3,625 and a maximum count of 24,976. In total, 22 distinct ARGs associated with seven antibiotic drug classes and four antibiotic resistance mechanisms: antibiotic efflux, antibiotic inactivation, antibiotic target alteration, and antibiotic target protection were identified (Supplementary Figure S2 and Supplementary Table S2).

**Figure 4:**
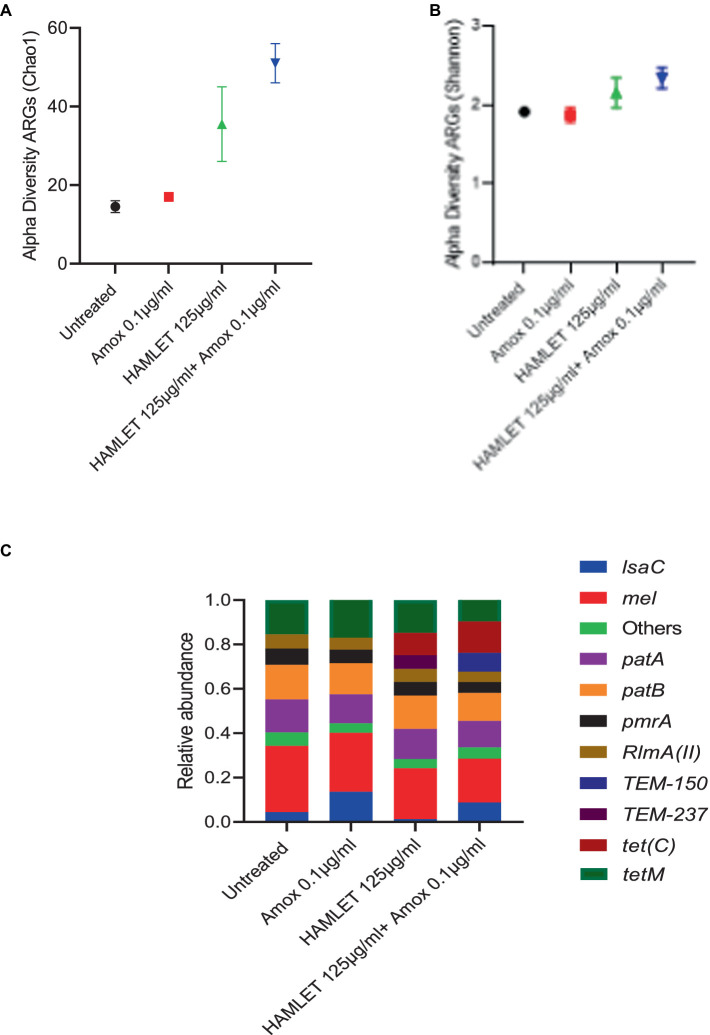
Impact of HAMLET alone or in combination with amoxicillin on oral resistome. **(A,B)** Alpha-diversity on ARG level measured by **(A)** Chao1 index indicates the total richness and **(B)** Shannon index indicates richness and evenness. **(C)** Stacked bar plots illustrate the relative abundance of all replicates for the 10 most abundant ARG’s. All results are based on two replicates.

Alpha-diversity, as measured by Chao 1 and Shannon indexes exhibited no major changes in the treatment groups when compared to the untreated control (Figures 4A,B). However, beta-diversity analysis revealed distinct clustering patterns of biological samples within each treatment group, signifying that each treatment group harbors a unique resistome (Supplementary Figure S2). ARGs associated with all four antibiotic resistance mechanisms were detected in all treatment groups, with the highest relative abundance observed in ARGs related to antibiotic target protection and antibiotic efflux (Supplementary Figure S2). The three most prevalent classes of ARGs in all treatment groups included fluoroquinolone, tetracycline, and macrolide-lincosamide-streptogramin (MLS). The proportion of beta-lactam ARG class appeared to be higher in the biofilms treated with HAMLET, as well as in those receiving the combined HAMLET and amoxicillin treatment, when compared to that in the negative control and the biofilms treated with amoxicillin alone (Supplementary Figure S2).

Regarding specific ARGs, *mel*, *patA*, *patB*, *pmrA*, *RImA(II)*, and *tetM* genes were detected in high abundance across all treatment groups (Figure 4C and Supplementary Table S3). Furthermore, the relative abundance of the *tet(C)* gene showed an increase in the HAMLET and HAMLET combined with amoxicillin-treated samples. For the beta-lactam antibiotic resistance genes, TEM genes were detected in all treated samples, although they comprised a low proportion of all ARGs.

## Discussion

With the treatment challenges of infections caused by biofilms and the growing global issue of antimicrobial resistance, there is an increased interest in identifying novel antimicrobials that can work in combination with antibiotics to lower the likelihood of microbial resistance (Kaneti et al., 2016; Basavegowda and Baek, 2022). Here, we investigated the combined usage of HAMLET and amoxicillin. HAMLET was specifically chosen due to its unique multi-targeted antimicrobial mechanism including inhibition of glycolytic pathways (Roche-Hakansson et al., 2019). Other promising properties of HAMLET include, lack of resistance development in studies with *S. pneumoniae* and *S. aureus* (Marks et al., 2012, 2013), and established low- or non-toxic profile in prior animal and human studies investigating its potential as an anticancer drug (Gustafsson et al., 2004; Mok et al., 2007; Puthia et al., 2014; Ho et al., 2017).

Our focus was on polymicrobial biofilm communities, an area that has received less attention compared to single bacterium in biofilms (Gabrilska and Rumbaugh, 2015; Joshi et al., 2021). Our results revealed that neither HAMLET nor amoxicillin individually in our comparative analysis had a significant effect on the overall cell viability of the polymicrobial community compared with the untreated control at the chosen concentrations. However, their combination resulted in a significant reduction in biofilm viability, indicating a possible synergistic effect. While acknowledging that not all bacteria are viable under culture conditions, our study employed also quantitative real-time PCR targeting the 16S rRNA genes, alongside biomass assessments. Both measures indicated higher values in treated samples, denoting a probable effect on other aspects of the biofilm, potentially attributable to the presence of dead cells, variations in 16S rRNA gene copy numbers, or differences in the extracellular matrix. These factors may make noteworthy contributions to biofilms. Furthermore, through metagenomic analysis, our data suggested that this combination may skew the polymicrobial community toward populations with potential probiotic effects, thereby representing a potential new approach on managing polymicrobial biofilms.

One of the most studied probiotic bacteria are lactobacilli. Several species in this genus have been shown to have beneficial effects, including improving gut and oral health, boosting the immune system, aiding in the digestion of lactose, and reducing the risk of certain infections (Kim and Gilliland, 1983; Kechagia et al., 2013; Chugh et al., 2020; Li et al., 2022; Mazziotta et al., 2023). *Lactobacillus fermentum* was the dominant species of lactobacilli in the non-treated control samples, comprising approximately 25% of the microbiome. These were practically absent in amoxicillin treated samples. It was therefore interesting that the combination of HAMLET and low-amoxicillin concentration in our study resulted in a microbiome dominated by lactobacilli. *L. crispatus*, in particular, was among those that increased in abundance from very low detected levels in the non-treated control and single treatments with HAMLET or amoxicillin to an average of more almost 60% with the combination of the agents. This species, although not commonly dominant in oral biofilms (Badet and Thebaud, 2008), is known for its probiotic and antimicrobial properties (Terai et al., 2020), and has been associated with oral health, particularly in the context of dental and periodontal diseases (Terai et al., 2015; Lin et al., 2018; Wang et al., 2022). These results highlight the potential of the combination of HAMLET and amoxicillin to modulate the composition of the microbiome toward a community enriched in probiotic bacteria, compared to samples treated with amoxicillin alone.

Although our study primarily aimed to investigate the combined effects of HAMLET and amoxicillin, the observations on HAMLET alone are also of relevance. We initially tested two concentrations of HAMLET for its potential effect on cell viability. From these, we chose the lowest concentration of HAMLET for the metagenomics studies to underscore its potential effect with amoxicillin. Although at this low dosage HAMLET alone had no discernible effects on the overall number of viable bacteria, the metagenomics analysis indicated potential changes in the microbiome composition with increased relative abundance of *S. salivarius*. It is possible that the changes by HAMLET, alone or in combination with amoxicillin, are a result of its influence on glycolytic pathways. Previous studies have shown that HAMLET binds to and inactivates two key glycolytic enzymes, fructose-bisphosphate aldolase and glyceraldehyde-3-phosphate dehydrogenase (GAPDH) (Roche-Hakansson et al., 2019). However, the mechanism of HAMLET’s antimicrobial effect is not yet fully understood.

In the case of antibiotics, it is known that at low concentrations bacteria can sense antibiotics as a stress, and rather than eliminating them, these low concentrations may promote stress responses that can favor overall survival (Roberts and Mullany, 2010; Ranieri et al., 2018; Preda and Săndulescu, 2019; Penesyan et al., 2020). This adds a level of complexity that needs to be considered in interpreting results using polymicrobial communities. Small changes in one microbial species caused by low concentrations of antimicrobials can trigger major ecological shifts in the community due to interdependent and non-linear interactions between microbial species.

In our study, we observed that *TEM* genes encoding beta-lactamases (Bush and Bradford, 2020) were present in samples exposed to HAMLET alone, amoxicillin alone or in combination with amoxicillin. While the increase in abundance of this gene in samples exposed to amoxicillin could potentially be linked to survival mechanisms in the presence of this beta-lactam antibiotic, it is difficult to explain the presence of this beta-lactamase in the HAMLET group alone. We also observed an increase in the abundance of *(tet)C* in both the groups treated with HAMLET alone and in combination with amoxicillin, despite tetracycline not being used in the study. Such increases in antibiotic resistance genes in response to the presence of low concentrations of antimicrobials are frequently reported in metagenomic studies and are often not solely attributed to the co-carriage of different antibiotic resistance genes in mobile genetic elements (Andersson and Hughes, 2014; Partridge et al., 2018; Shi et al., 2021). Instead, this phenomenon is reflective of the complex and intricate ecological dynamics within microbial communities, as discussed above.

Given the highly individual nature of human microbiomes (Gilbert et al., 2018), future studies exploring the role of modulatory agents will require large sample sizes to effectively predict which bacterial communities could benefit from the proposed interventions. However, metagenomic investigations are currently constrained by the substantial expense associated with deep sequencing and comprehensive metagenomic analyses. Despite these challenges—which limit the number of replicable studies—the high-resolution taxonomic profiling and the capacity to investigate important functional genes, including those for antibiotic resistance, underscores the significance of metagenomic approaches. Importantly, our metagenomic findings that the combination of HAMLET and amoxicillin led to an enriched presence of *L. crispatus* in the oral microbiome suggest a promising strategy for modulating microbial biofilm communities exposed to antibiotics, facilitating a healthier balance of probiotic bacteria.

## Data availability statement

The datasets presented in the this study can be found on the NCBI, accession number: PRJNA1046288.

## Ethics statement

The studies involving humans were approved by Norwegian National Regional Ethical Committee (REK20152491). The studies were conducted in accordance with the local legislation and institutional requirements. The participants provided their written informed consent to participate in this study.

## Author contributions

NB: Data curation, Formal analysis, Methodology, Resources, Software, Validation, Visualization, Writing – original draft, Writing – review & editing. AD: Data curation, Formal analysis, Investigation, Software, Validation, Visualization, Writing – review & editing. SS: Writing – review & editing. RJ: Writing – review & editing. AH: Conceptualization, Supervision, Writing – review & editing. FP: Conceptualization, Funding acquisition, Project administration, Supervision, Writing – original draft, Writing – review & editing.
