# Supplementary material for: HAMLET, a human milk protein-lipid complex, modulates amoxicillin induced changes in an ex vivo biofilm model of the oral microbiome
Source: Front Microbiol. 2024 Jul 4;15:1406190. doi: 10.3389/fmicb.2024.1406190 (PMC11254628; doi:10.3389/fmicb.2024.1406190)
Supplement: Supplementary file 1 [file Data_Sheet_1.pdf]

A

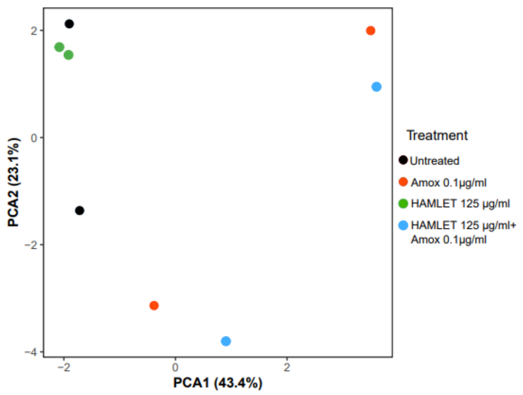

B

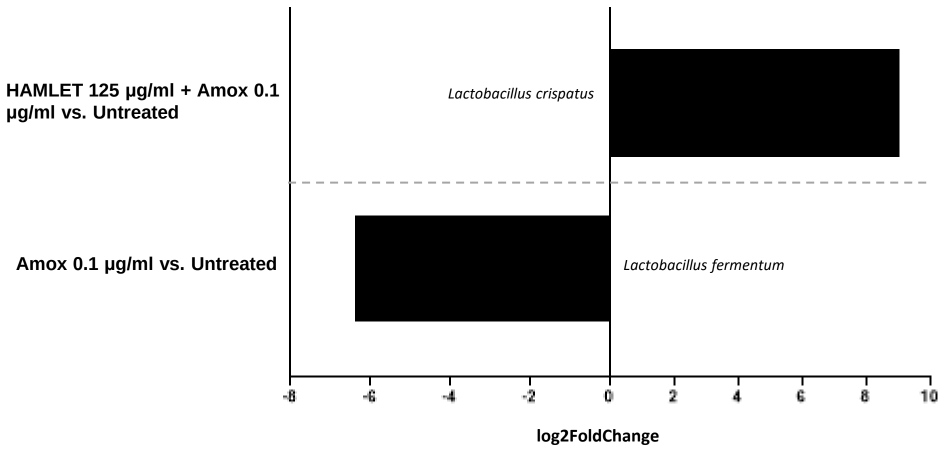

Supplementary Figure S1:Taxa with significantly different abundance upon treatment. (A) A principal component analysis plot (PCA) with Aitchison distance illustrating beta-diversity. (B) Bar charts illustrate the $\log_2$ fold change of taxa, adjusted for false discovery rate (FDR), *p*-values <0.05 (based on DESeq2).

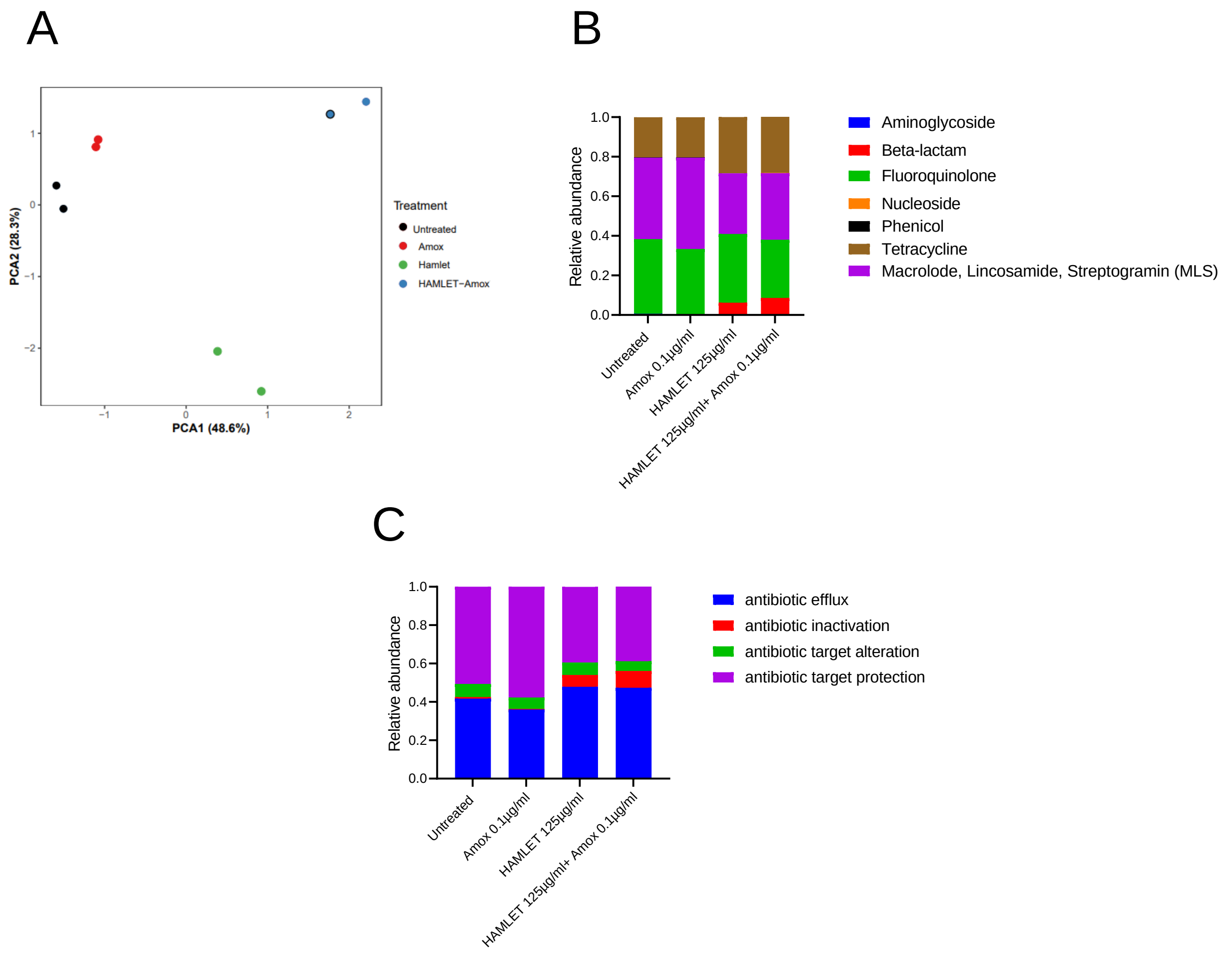

Supplementary Figure S2: Impact of HAMLET alone or in combination with amoxicillin on oral resistome. (A) Principal component analysis (PCA) ordination plot with Aitchison distance illustrating beta-diversity. (B-C) Stacked bar plots display the relative abundance of (B) all antibiotic mechanisms (C) all ARG classes.

Supplementary Table S1: Count table of shotgun metagenomic sequencing of biofilm community exposed to amoxicillin and HAMLET. Untreated [Control], Amoxicllin 0.1 µg/mL [Amox], HAMLET 125 µg/mL [HAMLET], HAMLET 125 µg/mL+ amoxicillin 0.1 µg/mL [HAMLET +Amox]

Feature.ID	Control-1	Control-2	HAMLET-1	HAMLET-2	HAMLET-Amox-1	HAMLET-Amox-2	Amox-1	Amox-2
Alloscardovia omnicolens	0	0	0	0	0	76	0	28499
Rothia mucilaginosa	1961	162	2236	2505	3103	3262	2854	1944
Gemella haemolysans	864	207	1859	1054	812	2303	1141	1384
Gemella morbillorum	72	245	220	0	0	323	0	0
Gemella sanguinis	3784	3336	6816	8158	5543	5165	7359	3383
Granulicatella adiacens	738	0	948	989	2324	4237	1185	1623
Granulicatella elegans	1974	852	2493	2041	1483	2309	1364	4051
Lactobacillus crispatus	0	6313	0	0	3041170	15822483	34739	111099
Lactobacillus fermentum	2109792	1496844	323095	2450459	0	0	0	52
Lactobacillus gasseri	0	0	0	0	565389	0	166561	0
Lactobacillus oris	0	0	0	0	0	0	455333	0
Lactobacillus paragasseri	0	0	0	0	2696443	0	989946	0
Lactobacillus pontis	0	0	0	0	0	0	1254423	0
Lactobacillus salivarius	0	0	0	0	0	73595	0	0
Streptococcus anginosus group	1041	1946	1239	360	3012	530	12438	435
Streptococcus australis	1796	340	929	1261	1424	666	2660	387
Streptococcus cristatus	4087	1980	14706	9690	13151	5537	13180	7877
Streptococcus gordonii	1916	0	0	2972	1499	293	1542	153
Streptococcus infantis	65845	21871	84989	80807	66429	65020	118229	62417
Streptococcus mitis	434727	236026	754729	704640	519234	831197	643245	1325673
Streptococcus oralis	150473	47337	305725	403706	159091	263555	250032	320118
Streptococcus parasanguinis	57479	17852	80618	91116	68530	75351	102708	99100
Streptococcus peroris	0	0	0	0	0	0	877	1304
Streptococcus pneumoniae	12647	7401	19247	17360	13095	28647	20291	50367
Streptococcus pseudopneumoniae	3764	554	3723	7863	5318	4886	5847	7494
Streptococcus salivarius	4878480	1467533	4981891	7042957	7750616	1210911	11557253	3338069
Streptococcus sanguinis	727	0	1322	1270	805	0	1927	601
Streptococcus sinensis	0	0	310	296	147	399	148	439
Streptococcus sp A12	3239	288	1282	2236	2749	859	3807	872
Streptococcus sp F0442	1164	87	1251	2075	1553	1020	2806	1003
Streptococcus sp HMSC034E03	8497	4040	21168	16858	18411	20618	20116	16534
Streptococcus sp HMSC067H01	10253	2230	16385	16878	11651	12312	22095	17377
Streptococcus sp HMSC070B10	1488	548	3964	3086	3339	0	3366	4447
Streptococcus sp HMSC071D03	9576	2249	17330	12750	10580	25313	26559	9651
Streptococcus sp HPH0090	270	0	1976	1762	537	211	2936	1660
Streptococcus sp M334	1137	1033	1297	286	0	838	715	3254
Streptococcus sp NLAE zl C503	2429	0	4290	4251	0	5721	5667	7738
Streptococcus vestibularis	429118	82052	121102	258666	200707	57107	364019	126149
Streptococcus viridans	0	0	0	489	0	0	0	0
Veillonella atypica	179	5205749	557	2617	0	19030	575	1853020
Veillonella dispar	0	426	0	1665	0	0	1027	219
Veillonella infantium	0	0	0	435	0	41	127	98
Veillonella parvula	0	245	0	260	0	0	93	1251
Escherichia coli	0	0	692	0	306	573	0	0

Supplementary Table S2: Relative abundance table of shotgun metagenomic sequencing of biofilm community exposed to amoxicillin and HAMLET. Untreated [Control], Amoxicllin 0.1 µg/mL [Amox], HAMLET 125 µg/mL [HAMLET], HAMLET 125 µg/mL+ amoxicillin 0.1 µg/mL [HAMLET +Amox]

Feature.ID	Control-1	Control-2	HAMLET-1	HAMLET-2	HAMLET-A	HAMLET-A	Amox-1	Amox-2
Alloscardovia omnicolens	0	0	0	0	0	0.00041	0	0.38461
Rothia mucilaginosa	0.02392	0.00188	0.03298	0.02246	0.02046	0.01759	0.01773	0.02623
Gemella haemolysans	0.01054	0.00241	0.02743	0.00945	0.00535	0.01242	0.00709	0.01868
Gemella morbillorum	0.00088	0.00285	0.00325	0	0	0.00174	0	0
Gemella sanguinis	0.04615	0.03875	0.10055	0.07314	0.03654	0.02785	0.04571	0.04565
Granulicatella adiacens	0.009	0	0.01398	0.00887	0.01532	0.02285	0.00736	0.02191
Granulicatella elegans	0.02407	0.0099	0.03678	0.0183	0.00978	0.01245	0.00847	0.05467
Lactobacillus crispatus	0	0.07332	0	0	20.04931	85.32222	0.21578	1.49937
Lactobacillus fermentum	25.73069	17.38546	4.76655	21.96969	0	0	0	0.0007
Lactobacillus gasseri	0	0	0	0	3.73	0	1.03459	0
Lactobacillus oris	0	0	0	0	0	0	2.8283	0
Lactobacillus paragasseri	0	0	0	0	17.77665	0	6.14904	0
Lactobacillus pontis	0	0	0	0	0	0	7.79184	0
Lactobacillus salivarius	0	0	0	0	0	0.39686	0	0
Streptococcus anginosus group	0.01269	0.0226	0.01828	0.00323	0.01986	0.00286	0.07726	0.00587
Streptococcus australis	0.0219	0.00395	0.01371	0.01131	0.00939	0.00359	0.01652	0.00522
Streptococcus cristatus	0.04985	0.023	0.21695	0.08688	0.0867	0.02986	0.08187	0.1063
Streptococcus gordonii	0.02337	0	0	0.02665	0.00988	0.00158	0.00958	0.00207
Streptococcus infantis	0.80304	0.25403	1.25383	0.72448	0.43794	0.35062	0.73438	0.84237
Streptococcus mitis	5.30186	2.74138	11.13434	6.31748	3.42312	4.48	3.99551	17.89095
Streptococcus oralis	1.83515	0.54981	4.51029	3.61944	1.04883	1.42121	1.55307	4.32023
Streptococcus parasanguinis	0.701	0.20735	1.18934	0.8169	0.45179	0.40633	0.63797	1.33743
Streptococcus peroris	0	0	0	0	0	0	0.00545	0.0176
Streptococcus pneumoniae	0.15424	0.08596	0.28394	0.15564	0.08633	0.15448	0.12604	0.67974
Streptococcus pseudopneumoniae	0.04591	0.00644	0.05493	0.0705	0.03506	0.02635	0.03632	0.10114
Streptococcus salivarius	59.49717	17.04502	73.49668	63.14391	51.09694	6.53	71.78778	45.04974
Streptococcus sanguinis	0.00887	0	0.0195	0.01139	0.00531	0	0.01197	0.00811
Streptococcus sinensis	0	0	0.00458	0.00265	0.00097	0.00215	0.00092	0.00592
Streptococcus sp A12	0.0395	0.00335	0.01891	0.02005	0.01812	0.00463	0.02365	0.01177
Streptococcus sp F0442	0.0142	0.00101	0.01845	0.0186	0.01024	0.0055	0.01743	0.01354
Streptococcus sp HMSC034E03	0.10363	0.04692	0.31229	0.15114	0.12138	0.11118	0.12495	0.22314
Streptococcus sp HMSC067H01	0.12504	0.0259	0.24173	0.15132	0.07681	0.06639	0.13724	0.23452
Streptococcus sp HMSC070B10	0.01815	0.00636	0.05848	0.02767	0.02201	0	0.02091	0.06001
Streptococcus sp HMSC071D03	0.11679	0.02612	0.25567	0.11431	0.06975	0.1365	0.16497	0.13025
Streptococcus sp HPH0090	0.00329	0	0.02915	0.0158	0.00354	0.00114	0.01824	0.0224
Streptococcus sp M334	0.01387	0.012	0.01914	0.00256	0	0.00452	0.00444	0.04392
Streptococcus sp NLAE zl C503	0.02962	0	0.06329	0.03811	0	0.03085	0.0352	0.10443
Streptococcus vestibularis	5.23346	0.95301	1.78659	2.31908	1.32319	0.30795	2.2611	1.70248
Streptococcus viridans	0	0	0	0.00438	0	0	0	0
Veillonella atypica	0.00218	60.46343	0.00821	0.02346	0	0.10262	0.00357	25.00789
Veillonella dispar	0	0.00495	0	0.01493	0	0	0.00638	0.00295
Veillonella infantium	0	0	0	0.0039	0	0.00022	0.00079	0.00132
Veillonella parvula	0	0.00284	0	0.00233	0	0	0.00058	0.01688
Escherichia coli	0	0	0.01021	0	0.00202	0.00309	0	0

Supplementary Table S3: count table of antimicrobial resistance genes (ARGs). Shotgun metagenomic sequencing of *ex-vivo* biofilm exposed to amoxicillin and HAMLET. Untreated [Control], Amoxicllin 0.1 µg/mL [Amox], HAMLET 125 µg/mL [HAMLET], HAMLET 125 µg/mL+ amoxicillin 0.1 µg/mL [HAMLET +Amox]

Feature.ID	Control-1	Control-2	HAMLET-1	HAMLET-2	HAMLET-Amox-1	HAMLET-Amox-2	Amox-1	Amox-2
gb|AB039845.1|+|25-1945|ARO:3000186|tetM [Erysipelothrix rhusiopathiae]	1436	581	1615	3252	2213	972	5314	2180
gb|AE005672.3|-|1980557-1982324|ARO:3000025|patB [Streptococcus pneumoniae TIGR4]	1391	640	2884	2037	1575	2586	2159	4051
gb|AE005672.3|-|1983115-1984810|ARO:3000024|patA [Streptococcus pneumoniae TIGR4]	1362	571	2629	1863	1511	2425	2130	3670
gb|AE007317.1|+|866210-867410|ARO:3000822|pmrA [Streptococcus pneumoniae R6]	637	318	1184	841	635	1019	1017	1670
gb|AF227521.1|+|3269-4487|ARO:3000616|mel [Streptococcus pyogenes]	2867	1018	3354	4177	4906	1655	6234	5544
gb|AF242872.1|+|2131-2878|ARO:3000375|ErmB [Enterococcus faecium]	48	17	105	99	87	69	124	108
gb|AM183304.1|+|208-1069|ARO:3001017|TEM-150 [Escherichia coli]	0	0	0	0	1212	1586	0	0
gb|AY043299.1|-|3984-5175|ARO:3000167|tet(C) [Aeromonas salmonicida]	0	0	2927	410	2071	2640	0	0
gb|AY712687.1|+|0-831|ARO:3002628|aad(6) [Streptococcus oralis] Partial	0	0	0	0	0	13	0	17
gb|CP004067.1|+|52914-53709|ARO:3002647|APH(3')-IIIa [Campylobacter coli CVM N29710]	0	0	0	0	21	22	0	0
gb|CP007593.1|+|2148923-2149772|ARO:3001301|RlmA(II) [Streptococcus pneumoniae]	569	260	1159	772	556	991	931	1464
gb|HM990671.1|+|5192-6671|ARO:3003112|lsaC [Streptococcus agalactiae]	526	51	210	229	2817	93	471	5559
gb|HQ652506.1|+|2099-3836|ARO:3004033|tetB(46) [Streptococcus australis]	180	54	226	152	236	139	291	190
gb|HQ652506.1|+|373-2098|ARO:3004032|tetA(46) [Streptococcus australis]	166	39	189	156	176	138	219	165
gb|KX000272.1|+|0-1740|ARO:3004035|tetA(60) [uncultured bacterium]	77	0	122	65	125	179	113	114
gb|KX000273.1|+|0-1740|ARO:3004036|tetB(60) [uncultured bacterium]	0	0	68	0	79	160	85	91
gb|M18896.2|+|206-2126|ARO:3000190|tetO [Campylobacter jejuni]	79	35	101	63	72	119	116	118
gb|M55620.1|+|0-660|ARO:3002687|catQ [Clostridium perfringens]	22	0	0	0	0	0	41	14
gb|NG_062250.1|+|100-961|ARO:3005268|TEM-237 [Escherichia coli]	0	0	1692	325	0	0	0	0
gb|U01945.1|+|373-916|ARO:3002897|SAT-4 [Campylobacter coli]	0	0	0	0	0	53	8	0
gb|U36911.1|+|1429-2290|ARO:3000979|TEM-116 [Staphylococcus aureus]	0	0	0	0	0	0	16	21
gb|V00618.1|+|150-945|ARO:3002644|APH(3')-IIa [Escherichia coli]	43	40	21	0	0	0	0	0
